# Comparison and Analysis of Body Composition of MMA Fighters and Powerlifting Athletes

**DOI:** 10.3390/jfmk10040388

**Published:** 2025-10-05

**Authors:** Jarosław Muracki, Kacper Olszewski, Arkadiusz Stanula, Ahmet Kurtoğlu, Gabriel Stănică Lupu, Michał Nowak

**Affiliations:** 1 Institute of Physical Culture Sciences, Department of Physical Culture and Health, University of Szczecin, 70-453 Szczecin, Poland; 2 Provita Medical Center, 44-240 Żory, Poland; 3 Institute of Sport Sciences, Jerzy Kukuczka Academy of Physical Education, 40-065 Katowice, Poland; 4 Department of Coaching Education, Bandırma Onyedi Eylül University, 10200 Balikesir, Türkiye; 5 Faculty of Movement, Sports and Health Sciences, “Vasile Alecsandri” University of Bacău, 600115 Bacău, Romania; 6 Department of Physical Culture Sciences, Collegium Medicum Named Dr. Władyslaw Biegański, Jan Dlugosz University in Czestochowa, 42-200 Częstochowa, Poland

**Keywords:** combat sports, training effects, phase angle, fat mass, muscle mass

## Abstract

**Background:** Mixed martial arts (MMA) is becoming increasingly popular and is developing dynamically in terms of training methods and number of participants involved, while weightlifting, powerlifting, and other kinds of strength disciplines are well established. In this study, the aim was to compare the body composition, as an anthropometric effect of training in MMA fighters and strength athletes, and then analyze and find reasoning for observed differences. **Methods:** Thirty-four young healthy male participants (body weight 84.9 ± 10.2 kg, body height 182.0 ± 6.8 cm, BMI 25.8 ± 2.51 kg/m^2^, tier 2/3 in McKay’s sports level classification) represented two groups: MMA (*n* = 17) and powerlifting athletes (STR, *n* = 17). The measured anthropometric characteristics were skeletal muscle mass (SMM), percentage of body fat (PBF), body fat mass (FM) and visceral fat mass (VFM). Phase angle (º) was measured as an indicator of tissue quality and we performed detailed investigations of soft fat-free tissue mass (SLM) and of fat mass in body parts separately in each lower and upper limb and trunk. **Results:** The groups did not differ in terms of body weight, height, BMI, SMM, PBF, FM, VFM, SLM in upper limbs and trunk, FM in the body parts, or the phase angle (all *p* > 0.05). The statistically significant differences were only observed in the SLM of both lower limbs (greater in STR, *p* < 0.05) but, after statistical correction with the Holm’s method, these parameters also did not show statistically significant differences despite high effect sizes. **Conclusions:** The MMA athletes do not differ significantly from strength training athletes in measured anthropometric parameters despite distinct differences in training methodology. The reasons for these observations need future research, combining anthropometric measurements with training and competing load monitoring.

## 1. Introduction

### 1.1. MMA Training Characteristics

Mixed martial arts (MMA) has become a widely popular combat sport discipline worldwide [1]. Regarding its global growth, we might consider the creation of the Ultimate Fighting Championship (UFC), which held its first pay-per-view event in 1993. MMA implements both striking and grappling techniques derived from many other combat sports, which consequently makes MMA athletes very versatile [2]. To this day, MMA’s complexity and unpredictability make it difficult to compose an ideal and universal training regimen [3,4]. The same can be said about matching proper prehabilitation and rehabilitation protocols. Advanced and experienced MMA athletes should possess a wide range of hard-to-quantify locomotor and kinesthetic skills, including dynamic and static balance, the ability to combine and adjust movements, and time and space orientation [5]. However, when it comes to primary biomotor skills, which can be easier to test and define, the final recommended parameters might differ from athlete to athlete when taking into account their fighting style [3]. The skill dependency may also vary in different weight categories [6,7]. In general, MMA has a demand for high relative strength and power [8,9,10,11] and the full range of energy systems, both aerobic and anaerobic [8,9,10,12].

Tudor Bompa & Carlo Buzzichelli originally presented a model depicting the relationship between strength training and energy systems. However, the complexity of MMA, which consists of both striking and grappling, makes it extremely difficult to create a universal parameter model for an MMA athlete. The model [13] involved interrelationships between biomotor skills as well. While in strength sports, the demands on motor skills are narrower, MMA is distinguished by its multitude and alternating usage, which brings up the question—is there any quantifiable optimal morphological/architectural/structural feature composition for an MMA athlete?

There are indications that the implementation of proper strength and conditioning training program focusing on maximal strength and power can increase general physical efficiency of MMA athletes [1,2] which may suggest that strength-focused discipline athletes can be a good reference group to compare MMA fighters with when it comes to anthropometric features.

### 1.2. Powerlifting Characteristics

Strength sports group can be divided into many disciplines, with the most common ones being bodybuilding, powerlifting, and Olympic Weightlifting [14]. There are many others, including strongman or calisthenics. Hybrid athletes can also be taken into consideration, for example, CrossFit [15] or OCR (Obstacle Course Racing) runners. The most popular of the aforementioned disciplines in the general population is bodybuilding; however, it is mostly performed on an amateur/non-competition level [16,17,18]. Powerlifting consists of only three lifts: squat, bench presses and deadlifts. This is why, in this study, we examined powerlifters (STR), who are among the most straight-lined strength-focused athletes [19].

In MMA, training sessions are based on various elements, including grappling, striking, and training drills that focus on energy systems. Powerlifters tend to train and operate in a specific and confined way. They are likely to train in work sets with few repetitions to maintain high force development. This, consequently, allows them to prioritize specific muscle parameters more than others: maximum strength, power or hypertrophy. Thus, in powerlifting, a training regime can be conducted with minor or no priority for muscle endurance and/or energy systems depending on currently desired adaptations.

Thomas Baechle, along with Roger Early, created a model of the repetition maximum continuum, which describes how different structural, morphological, and architectural muscle parameters respond to various stimuli [20]. In powerlifting, many training plans and periodization are based around subjective work effort up to the point of muscle failure [13]. The most commonly used methodologies are RPE and RIR, where RPE refers to the rate of perceived exertion scale (based on another Borg Scale) [21] and RIR represents repetitions in reserve [22]. There are also non-subjective methods in use, like velocity-based training (VBT) using accelerometers or LPTs and others, electromyography (EMG) [23], and many others. While powerlifting can be easily placed and performed using the aforementioned methods, this does not apply to MMA. Another aspect would be the speed of movement itself. While in powerlifting, speed is not a decisive factor, in combat sports, it is extremely important. Thus, the training regime for the competitive-level athlete will differ for both disciplines in this matter as well. It is well represented by the force–velocity curve [24,25], where powerlifters will focus on left, high-force, low-velocity movements, while MMA athletes operate in the whole range.

In STR, it is relatively easy to determine requirements and dependencies between main biomotor skills (strength–speed–endurance), while in MMA, it may strongly depend on the style and preferred tactic of an individual athlete. In this context, comparing the effects on body composition of these disciplines, which have different training and biomechanical characteristics, may also help to understand the physiological outcomes of these characteristics.

### 1.3. Phase Angle

Phase angle (PhA) is a biophysical parameter that reflects cell membrane integrity and intracellular mass state, expressing the angle between resistance and reactance to electrical current passing through cells. Phase angle (PhA) is a useful biomarker that can be used to describe general body cell health. It has been strongly linked to health status, physical performance, and potential mortality rate as well [26,27,28,29,30,31,32,33]. There are two common methods used to determine this factor through body impedance: bioelectrical impedance spectroscopy (BIS) and bioelectric impedance analysis (BIA), of which BIA has gained increasing popularity because of its low cost, simplicity, and noninvasiveness. BIA also has wider body composition examination capabilities, including whole-body and partial assessments that divide the body into smaller parts, such as the torso, lower limbs, and upper limbs [29]. Taking all this into account, there is still a lack of proper, clearly defined reference values for many of the sport disciplines [34].

As stated before, there are not many studies depicting values or potential differences in bioimpedance between combat sports. MMA is becoming more and more popular, yet it is still relatively young and undiscovered, thus making it the perfect sport to focus on. Powerlifting, as a very straightforward strength-focused discipline, makes for a perfect control group to compare anthropomorphic characteristics for muscle mass quality and body composition, since both have weight class restrictions.

### 1.4. Aim of the Study

A review of the literature reveals that MMA and STR athletes differ in their training and competition content, biomechanical behavior, and the energy systems they utilize. Very few studies have focused on how body composition is shaped in these different disciplines in terms of their distinct content. In this context, the main goal of this study was to test the hypothesis that MMA athletes have as high tissue quality and exquisite body composition as elite strength athletes, using powerlifters as control group. Secondly, we aimed to determine if there were any significant differences in MMA athletes’ body composition between left and right limbs, as this has been studied in other combat sports [35,36,37,38,39]. Findings can also help to estimate the optimal anthropometrical profile of an MMA athlete. Additionally, obtained results might serve as reference data for future research.

## 2. Materials and Methods

### 2.1. Study Design

In this study, two groups of atlethes (MMA and STR) were investigated for antrophometric measurements. The measurements were performed once. An a priori power analysis was conducted in G*Power 3.1.9.7 (Kiel University, Germany) [40] for a two-tailed independent samples t-test comparing studied variables between two independent groups. We assumed a medium effect size (d = 0.50), α = 0.05, and a desired power (1 − β) = 0.80. Under these assumptions, G*Power indicated that 64 participants per group (total N = 128) would be required to detect a medium-sized difference. Given our actual sample sizes (*n*_1_ = 17, *n*_2_ = 17), the achieved power was approximately 0.57, suggesting the study was underpowered to detect a medium effect on studied variables.

### 2.2. Subjects

As we decided to recruit only high-level athletes according to strict exclusion and inclusion criteria, there were initially 40 participants recruited. Finally, 34 young (24 ± 3.5 years) healthy male participants (body weight 84.9 ± 10.2 kg, body height 182.0 ± 6.8 cm, BMI 25.8 ± 2.51 kg/m^2^) were involved and finalized all the study procedures. The participants represented two groups: MMA fighters (*n* = 17) and powerlifters (STR, *n* = 17). According to the McCay classification, both groups represent tier 2/3 [41]. The comparative analysis of the anthropometric data of these groups is presented in Table 1. The MMA fighters trained in mixed martial arts, with a history of other combat sports, before joining the MMA sport club. They were divided into groups according to the UFC weight divisions: welterweight (up to 77.1 kg)—6, middleweight (up to 83.9 kg)—4, light heavyweight (up to 93.0 kg)—3, heavy weight (up to 120.2 kg)—4. The STR group participants had a history of general strength training for 5 years and competed in powerlifting tournaments and championships for the same period. They represented the following weight categories according to the International Powerlifting Federation (IPF): up to 74 kg—3, up to 83 kg—3, up to 93 kg—5, up to 105 kg—5, up to 120 kg—1.

Although there were differences in the weight classification, the number of participants in each category was similar in both groups, and there were no statistically significant differences in body mass, body height, and BMI between groups (Table 1). The measurements were conducted between 5 and 7 days after the end of the main season for both groups.

The inclusion criteria for both groups were the same. Athletes had a minimum of 5 years of training, which was completed with a training frequency of a minimum of 3 times a week. All were males, aged between 20 and 30. None had a history of serious injuries or injuries eliminating them from training participation for more than 3 days in the last year.

The exclusion criteria included psychodietetic problems, using extraordinary diets other than the normal diet, or a history of using anabolic steroids. Gastrological problems or a history of cancer or other serious diseases would also exclude potential participants.

All participants were informed about the study’s conditions and that they could withdraw at any stage of the project without consequences and signed written informed consent to participate. The research was conducted in accordance with the Declaration of Helsinki and received approval from the Ethics Committee of the Polish National Council of Physiotherapists (no. 24/04/2023).

### 2.3. Procedures

Body composition was measured using the InBody 770 MF-BIA system (InBody Co., Seoul, Republic of Korea 2020), calibrated according to manufacturer instructions. Participants underwent testing in the morning after an overnight fast (≥8 h), with hydration status confirmed via urine specific gravity. Measurements were conducted in a standardized upright posture, with limbs not in contact with the torso. The InBody 770 has been demonstrated to yield excellent test–retest reliability (ICC ≥ 0.98–0.99 for fat mass, fat-free mass, and body fat percentage), and stable day-to-day repeatability (ICC ≥ 0.973–1.000 for regional and whole-body measures) [42].

### 2.4. Statistical Analysis

Descriptive statistics for each variable included the arithmetic mean (M), standard deviation (SD) and median (Me), together with measures of distributional shape—skewness (Sk) and excess kurtosis (Ku). To screen for univariate normality, values of |Sk| [43] and |Ku| within ±2 were deemed acceptable, in accordance with George and Mallery’s (2010) recommendation that such bounds approximate a normal distribution; variables exceeding these limits were subjected to the Shapiro–Wilk W test to confirm non-normality [44]. Between-group comparisons of powerlifting (STR) and mixed martial arts (MMA) athletes were carried out using independent-samples Student’s t-tests, testing H_0_: μ_str_ = μ_mma_ versus H_1_: μ_str_ ≠ μ_mma_. For each contrast, we report the mean difference (ΔM), its 95% confidence interval (±95% CI), and the proportional change (Δ %). To control the family-wise error rate due to multiple testing, *p*-values were adjusted using Holm’s step-down procedure, applied within pre-specified families of outcomes (e.g., anthropometric, body composition, or segmental parameters). Statistical significance was determined based on adjusted *p*-values (*p*.adj). Effect magnitude was quantified by Cohen’s d, interpreted according to Cohen (2013) as negligible (d < 0.20), small (0.20 ≤ d < 0.50), medium (0.50 ≤ d < 0.80) or large (d ≥ 0.80) [43], with corresponding 95% CIs; an interval excluding zero denotes a population-level effect. All hypothesis tests were two-tailed at α = 0.05. Statistical computations of descriptive, inferential and effect-size estimates were performed in STATISTICA 13.0 PL (TIBCO Software Inc., San Ramon, CA, USA, 2017), and graphical visualizations were generated in R 4.4.1 (R Core Team, Vienna, Austria, 2024) using ggplot2 3.5.1 [45]. The results of the research will be presented accordingly.

## 3. Results

### 3.1. Assessment of Basic Somatic Features (Body Mass and Height, BMI)

Table 1 presents detailed results of the comparative analysis of body mass, height, and body mass index (BMI) in the groups practicing powerlifting (STR) and MMA. The STR group showed higher body mass (88.4 ± 11.47 kg vs. 81.8 ± 10.83 kg), body height (182.4 ± 6.26 cm vs. 181.0 ± 7.16 cm), and BMI (26.49 ± 2.26 kg/m^2^ vs. 24.98 ± 3.12 kg/m^2^) values compared to the MMA group. However, none of these differences reached the level of statistical significance.

**Figure 1:**
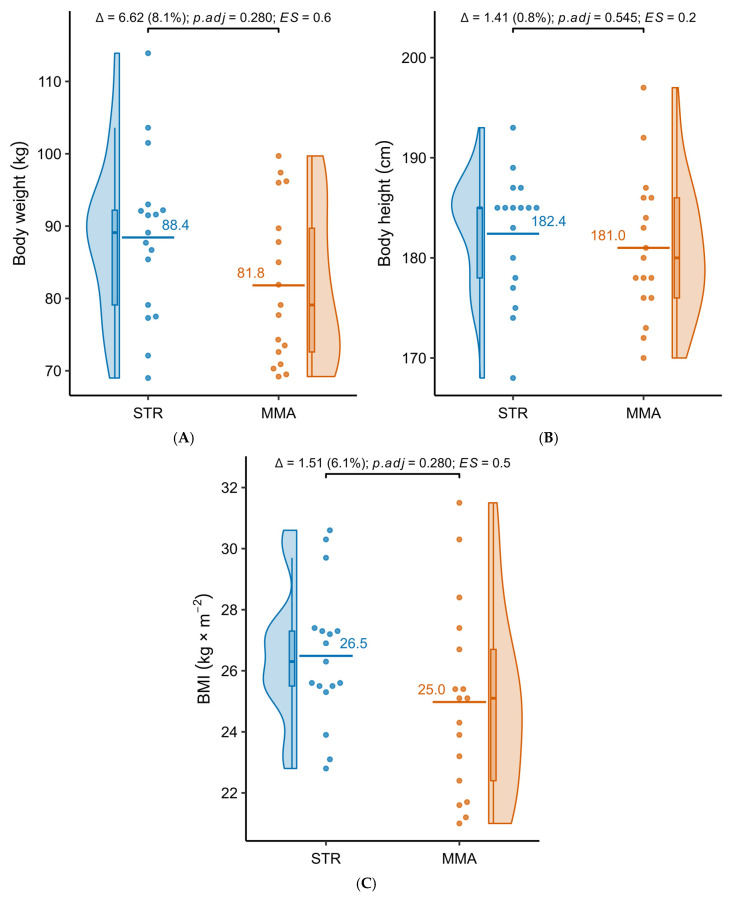
Distributions of body mass (**A**), body height (**B**) and BMI (**C**) in the groups of people practicing powerlifting (STR) and mixed martial arts (MMA).

Figure 1A–C present detailed distributions of the analyzed variables.

### 3.2. Assessment of Body Composition (Skeletal Muscle Mass, Percentage of Fat Mass and Visceral Fat Mass)

Table 2 presents detailed results of the comparative analysis of selected body composition parameters in the groups practicing powerlifting (STR) and MMA. The STR group showed higher skeletal muscle mass (42.65 ± 5.77 kg vs. 39.03 ± 4.94 kg). However, after adjustment for multiple testing, this difference did not reach the level of statistical significance (*p*.adj = 0.236). The percentage of body fat was slightly lower in STR (13.84 ± 2.49%) than in MMA (14.68 ± 4.08%), while body fat mass (12.23 ± 2.61 kg vs. 12.14 ± 4.11 kg) and visceral fat mass (1.42 ± 0.38 kg vs. 1.41 ± 0.60 kg) were very similar between groups. None of these differences were statistically significant.

**Figure 2:**
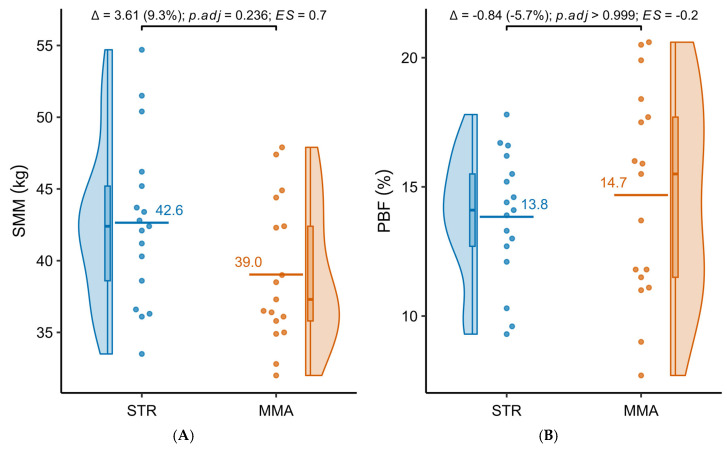

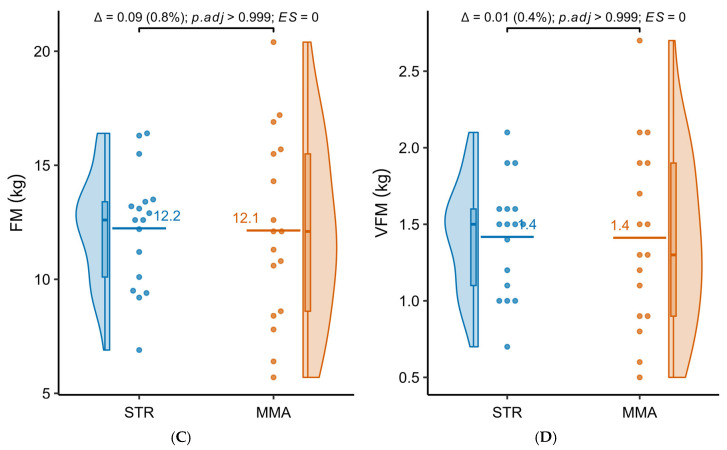
(**A**–**D**) Distributions of the analyzed body composition variables of the subjects practicing powerlifting (STR) and MMA.

Figure 2A–D present detailed distributions of the analyzed variables.

### 3.3. Assessment of Lean Soft Tissue Mass (In Body Parts, i.e., Upper and Lower Limbs and Trunk)

**Figure 3:**
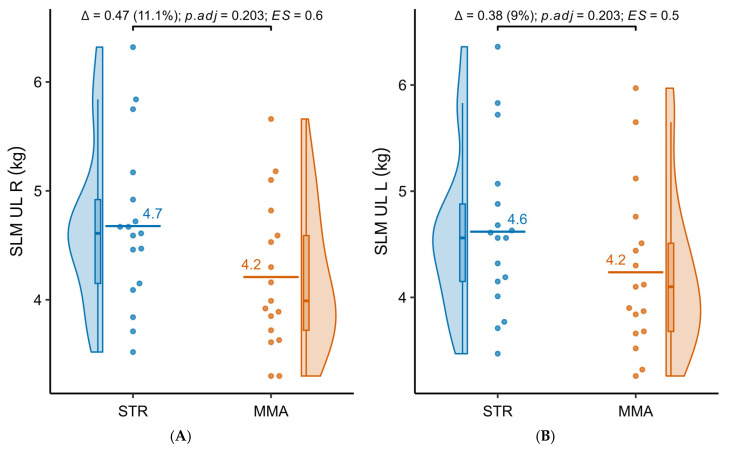

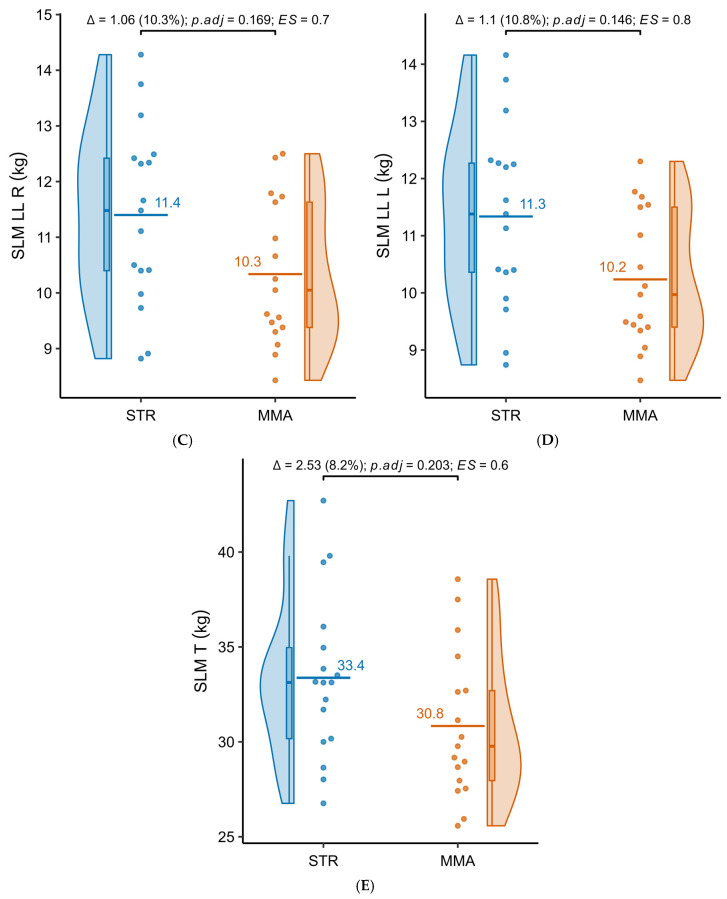
(**A**–**E**) Distributions of lean soft tissue mass (SLM) in different body parts of the subjects practicing strength sports (STR) and MMA. UL—upper limb, LL—lower limb, L/R—left/right, T—trunk.

Table 3 presents the results of the comparative analysis of lean soft tissue mass in the groups practicing powerlifting (STR) and MMA. In the upper limbs and trunk, the STR group showed higher values ((right upper limb: 4.68 ± 0.76 kg vs. 4.21 ± 0.68 kg (*p*.adj = 0.203), d = 0.63; left upper limb: 4.62 ± 0.78 kg vs. 4.24 ± 0.77 kg, (*p*.adj = 0.203); trunk: 33.37 ± 4.30 kg vs. 30.84 ± 3.91 kg, (*p*.adj = 0.203, d = 0.60)). These differences did not reach the level of statistical significance, although those for the right upper limb and trunk indicated medium effect sizes. In the lower limbs, the STR group also had higher lean mass (right: 11.40 ± 1.61 kg vs. 10.34 ± 1.29 kg, (*p*.adj = 0.169), d = 0.71; left: 11.34 ± 1.59 kg vs. 10.23 ± 1.18 kg, (*p*.adj = 0.146), d = 0.77). However, after correction for multiple testing, none of these differences remained statistically significant, despite the presence of medium effect sizes. Figure 3A–E present detailed distributions of the analyzed variables.

### 3.4. Assessment of Fat Mass (In Body Parts, i.e., Upper Limb, Lower Limb and Trunk)

**Figure 4:**
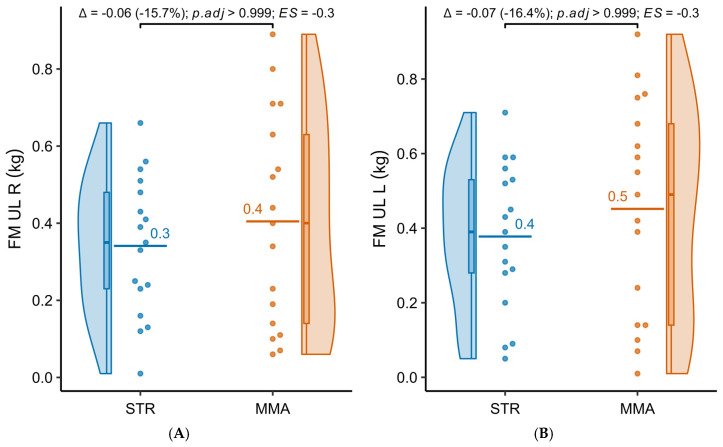

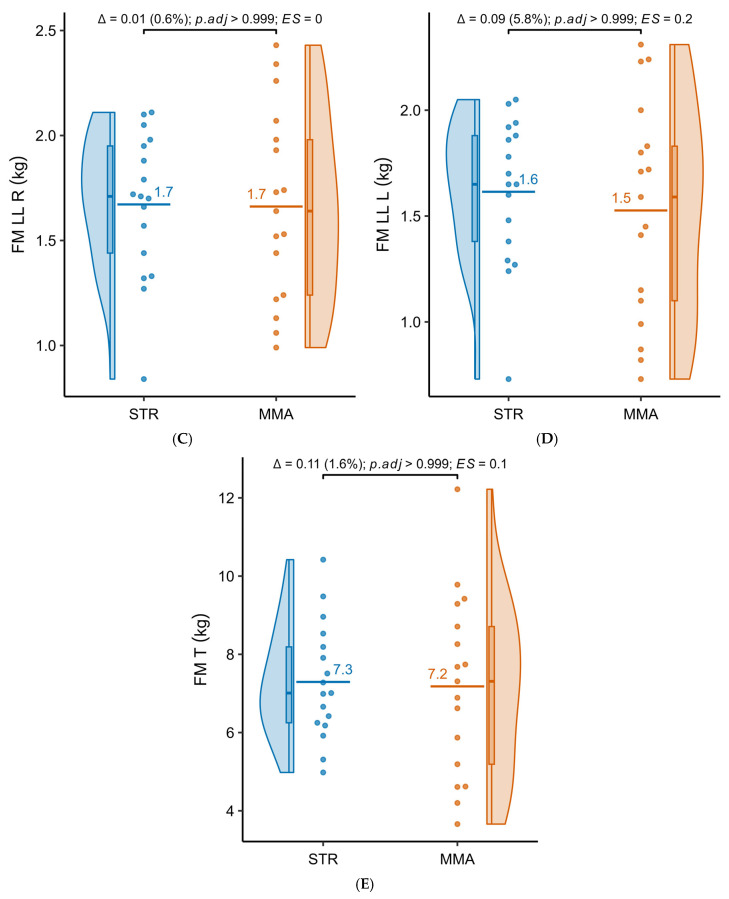
(**A**–**E**) Distribution of fat tissue mass in different body parts of the examined individuals practicing powerlifting (STR) and MMA. FM—fat mass, UL R/L—upper limb right/left; T—trunk; LL R/L—lower limb right/left.

Table 4 presents the results of the comparative analysis of adipose tissue mass in the groups practicing powerlifting (STR) and MMA. The values were generally similar between groups. In the upper limbs, STR showed slightly lower fat mass (right: 0.34 ± 0.18 kg vs. 0.41 ± 0.28 kg; left: 0.38 ± 0.20 kg vs. 0.45 ± 0.29 kg), while in the trunk (7.29 ± 1.48 kg vs. 7.18 ± 2.32 kg) and lower limbs (right: 1.67 ± 0.35 kg vs. 1.66 ± 0.46 kg; left: 1.61 ± 0.35 kg vs. 1.53 ± 0.52 kg), the results were very similar. None of these differences reached statistical significance after adjustment for multiple testing (Holm; all *p*.adj = 1.000). Figure 4A–E present detailed distributions of the analyzed variables.

### 3.5. Phase Angle Assessment as a Tissue Quality Indicator

**Figure 5:**
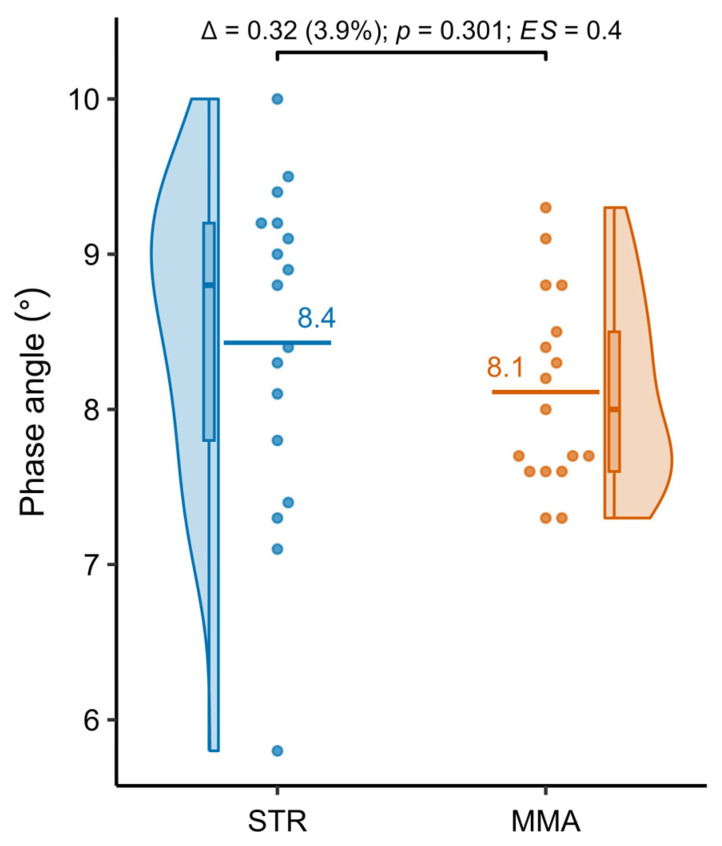
Distribution of phase angle values of the examined individuals practicing powerlifting (STR) and MMA.

Table 5 presents the results of the comparative analysis of the phase angle (tissue quality indicator) in the groups practicing powerlifting (STR) and MMA. The STR group showed slightly higher values (8.43 ± 1.07° vs. 8.11 ± 0.62°), but the difference was not statistically significant. Figure 5 presents detailed distributions of the analyzed variables.

## 4. Discussion

The results obtained in this study indicate that powerlifters have higher body weight (88.4 ± 11.47 kg vs. 81.8 ± 10.83 kg) and BMI (26.49 ± 2.26 kg/m^2^ vs. 24.98 ± 3.12 kg/m^2^) values compared to MMA athletes. Although there is no significant difference between these results, a moderate effect size explains the formation of morphological adaptations specific to strength sports. In a previous study conducted on UFC athletes, BMI values ranged from 22.5 to 25.8 kg/m^2^ according to weight classes [7]. Camarco et al. (2019) reported an average BMI of 24.48 ± 3.63 kg/m^2^, which was quite close to the MMA group in this study [46]. Similarly, Bueno et al. reported an average BMI of 26.0 ± 3.2 kg/m^2^ in professional MMA athletes. However, the body fat percentage in the MMA group in this study was lower (14.68 ± 4.08%) [47]. In another study by Marinho et al., the BMI of MMA athletes was reported as 26.0 ± 3.3 kg/m^2^, and the body fat percentage as 13.4 ± 5.6% [48]. Schick et al. (2010) reported a PBF value of 11.7 ± 4.0%, highlighting the importance of weight control and lean body mass ratio in MMA due to the lower fat percentage [49]. This situation draws attention to the variable body composition during training and competition periods in MMA athletes due to weight management [50]. The higher body mass and BMI in powerlifters are likely due to the inclusion of high-resistance exercises in their training. This is because muscle mass in these individuals is more closely associated with maximal strength production than functional performance. In MMA athletes, however, training shows a broader distribution between aerobic and anaerobic energy systems, increasing metabolic demand and thereby potentially reducing body fat percentage. It should be emphasized that the speed of movement execution is not a key target parameter in powerlifting. However, it plays a big role in terms of potential hypertrophy and musculoskeletal system performance. Rossi et al. (2024) [51] conducted a study on 34 young females comparing effects of velocity-based training (VBT) and ordinary training based on the percentage of maximal effort. They found that the VBT group showed higher maximal strength growth in comparison to the test group. This further shows the adaptive potential of speed and acceleration related training, which can be found in combat sports among others.

Powerlifters were characterized by a higher BMI (constituting +6.1% relatively), which was a consequence of higher SMM (+9.1% relatively) and SLM (ranging from 8.2% to 11.1% relatively, depending on the body part). This is probably a result of limited training variety in strength sports focused on the narrow force–velocity curve portion.

PBF% was higher in MMA athletes (by 5.7% relatively); however, FM was similar (0.8% value difference between), which again was related to higher SMM and SLM in powerlifters. This result is baffling. In MMA, there is a definite greater priority on energy systems. This higher demand will result in more aerobic and anaerobic work in the training regimen. Aerobic work has a relatively high energy consumption per unit of time. Anaerobic work, especially High-Intensity Interval Training (HIIT), is associated with Enhanced Post-exercise Oxygen Consumption (EPOC). EPOC is linked to an elevated resting metabolic rate, which causes increased energy expenditure after physical effort (for example, a training session) [52,53]. Taking all this into account, it would suggest that MMA fighters with more conditioning elements in their regimen should exhibit lower body fat mass and percentiles. On the other hand, there is a conviction in part of the combat sports environment that there might be a correlation between FM/PBF% and potential trauma prevention, meaning that FM serves as an extra cushion [54] and/or provides resistance [55]. This, however, needs further examination, as no clear data confirms this theory.

### 4.1. Phase Angle

There are many studies with bioelectrical impedance phase angle (PhA) measurements; however, none contain MMA or powerlifting [56,57,58]. The only option is to compare the present research results on MMA with other disciplines. The mean PhA in the powerlifters group was 8.43 ± 1.07°, and in the MMA group it was 8.11 ± 0.62°. The difference between the groups was 0.32° (±95% CI: −0.30, 0.94), representing a difference of 3.9%. The observed difference was not statistically significant (*p* = 0.301). The calculated Cohen’s d statistic value was 0.35 (±95% CI: −0.44, 1.05), indicating a small effect size. The fact that the PhA is significantly high in both groups is known to be an important predictor of energy expenditure during rest in elite athletes [58]. The relatively high phase angle observed in both groups in the present study may reflect the good cellular integrity and hydration status often seen in athletes competing at elite levels [59]. On the other hand, studies suggest that phase angle is a marker for muscle quality and cellular health, with higher values associated with greater muscle mass and lower inflammation [60]. The team from University of Naples conducted research on elite sportsman from different sport disciplines. They registered PhA values for the following disciplines: cycling (8.31 ± 0.79), water polo (8.11 ± 0.49), karate (7.59 ± 0.60), ballet dance (7.75 ± 0.53), boxing (8.57 ± 0.65), and running (7.60 ± 0.38). O. Di Vincenzo et al. evaluated PhA in invasion sports, with a median result of 7.7% for PhA. For individual sports, they registered the following PhA values: basketball, 8.1 ± 0.8; football, 8.1 ± 0.8; futsal, 8.3 ± 1.2; handball, 8.1 ± 0.6; rugby, 7.4 ± 0.6 [56]. F. Campa et al. examined 1658 male athletes on a national level from over 20 different disciplines, with the median PhA equal to 7.6 or 7.7 depending on the activity [57]. When the results of this study were examined, it was determined that the PhA value of MMA athletes was at an adequate level [58]. Therefore, despite differences in muscle mass and training profiles, the similarity in PhA in powerlifters and MMA athletes suggests that optimal intracellular balance is maintained in both branches and reflects a high level of physical preparation for both branches.

### 4.2. Body Composition

Asymmetries in body composition between left and right limbs, which can be found in other combat sports like BJJ or Judo [35,39], were not present in this study’s MMA group participants. This may be confirmation of the versatility required in MMA.

One of the important findings in our study was that although powerlifters had more skeletal muscle mass, there was no significant difference in fat mass and phase angle in both groups. This may be attributed to the fact that MMA athletes have a relatively more favorable body composition due to the careful planning of weight management strategies before competition [61]. Furthermore, muscle mass has the property of metabolic regulation in the body, which may explain why increased muscle mass in strength athletes is not directly associated with a decrease in fat mass, since hypertrophic muscle tissue may prioritize force and power production over metabolic efficiency [62]. From a biomechanical point of view, higher SLM values in strength athletes, especially in the lower extremities, indicate an adaptation to the mechanical demands of high-load, stable, and closed kinetic chain movements [63,64]. In contrast, MMA sports emphasize more dynamic, multi-planar movements, explosive force production, and energy conservation through the kinetic chain [63]. This difference in movement pattern may partially explain the lower SLM distribution in MMA athletes.

An important finding of the present study was that lower extremity soft lean tissue percentage was higher in powerlifters compared to MMA athletes. This difference in the lower extremities may be due to the fact that powerlifters include specific exercises for hypertrophy of the lower extremity muscles in their training plans [63]. In this context, MMA athletes had lower SLM in the lower limbs than the powerlifting group. This suggests that lower extremity hypertrophy in strength-trained individuals occurs due to the desire to develop maximal strength. Increased neuromuscular capacity, together with musculoskeletal hypertrophy due to high mechanical loading in strength sports, may be the main reason for this increase in SLM in the lower limbs [61]. In addition, training for specific muscles or muscle groups (squats, deadlifts, etc.) makes important contributions to the formation of this hypertrophic response [63]. Although upper extremity and trunk SLM values were higher in powerlifting group, the lack of statistical significance between the two groups may be due to the fact that MMA athletes focus more on speed, agility, and flexibility due to their training based on functional mobility and applying different exercise plans together. Therefore, MMA athletes develop different energy systems and more complex neuromuscular adaptations [2].

When the upper extremity fat mass of the participants was analyzed, powerlifters had lower fat mass than MMA athletes, but these differences were not significant. This may be due to strength athletes incorporating more high-resistance and repetitive loads into their training. This supports regional hypertrophy and suppresses fat mass [65]. In MMA athletes, training content typically consists of high-intensity intervals, complex technical movements, and isometric resistance exercises. This suggests that MMA athletes have a more homogeneous distribution of fat mass in the lower and upper extremities [66]. Although segmental fat percentages appear to be relative in athletes, previous studies have suggested that this difference may have a cushioning effect against injuries [67]. Additionally, the similarity in body fat mass between the two groups indicates that the muscle groups responsible for core region stabilization are activated in a similar manner in both sports and that there is a similar need for connection to the lower and upper extremities in both sports [68]. As a result, these differences in segmental fat mass are thought to be due to physical adaptations specific to the nature of the sport and physical adaptations that develop depending on the training structure, which may have important effects on individual performance outcomes.

There are visible differences in test values between studies including MMA athletes in both in anthropometry and bioimpedance. This is most probably due to differences in inclusion and exclusion criteria for participants. The present study placed a big priority on conducting research with high-class athletes. In comparison with different disciplines, MMA athletes turned out to be at least in the upper ranges when it comes to PhA, as an indicator of indirect tissue quality and cell health.

## 5. Conclusions

The findings of the study demonstrated minor somatic differences between powerlifter athletes and MMA fighters, with SMM showing a 9.3% difference, PBF showing a 5.7% difference, FM showing a 0.8% difference, and VFM showing a 0.4% difference (higher in the powerlifters). For SLM, the differences between STR and MMA groups were, respectively, 11.1% for right upper limb, 9.0% for left upper limb, 8.2% for trunk, 10.3% for right lower limb, and 10.8% for left lower limb. Although the highest magnitude involved FM in upper limbs, with a 15.7% difference for the right upper limb and a 16.4% difference for the left upper limb, only the results of SLM in both lower limbs showed statistically significant differences (*p* = 0.042, ES 0.71 for the right lower limb and *p* = 0.029) between the STR and MMA groups where the powerlifter athletes had higher results (11.4 ± 1.61 vs. 10.34 ± 1.29 in the right lower limb and 11.34 ± 1.59 vs. 10.23 ± 1.18 for the left lower limb). However, after Holm’s correction, both differences did not reach the level of statistical significance with *p*.adj values of *p* = 0.169 and *p* = 0.146 accordingly, despite the high ES values of 0.71 for the right and 0.77 for the left lower limb.

While the lack of significant differences between right and left upper and lower limbs in the powerlifter athletes were expected, the results showed that there were no statistically significant differences between right and left upper and lower limbs in the MMA group, which underscores that MMA requires very versatile athletes, using both sides of the body very evenly.

In both powerlifting and MMA groups, athletes had high PhA values (STR 8.43 ± 1.07° and in MMA 8.11 ± 0.62°) which is an indication of good cell and tissue health.

## 6. Practical Implications

1. MMA athletes exhibit tissue quality and body composition characteristics similar to those of strength athletes, which may serve as a guideline for planning a well-fitting training regimen that includes components focused on peak force, maximum strength, and isometric strength.
2. The lack of existing significant anthropometric asymmetries in MMA fighters can be used for guidance in strength and conditioning programming, namely, to point out the necessity to focus on bilateral exercises when it comes to strength and/or hypertrophy exercises.
3. The PhA value for strength and MMA athletes defined in this study can be used as references for nutritionists, coaches, and athletes themselves to assess whether the current health status of the athlete is satisfactory.

## 7. Limitations of the Study

MMA is a relatively new combat sport; it only appeared in the 1990s. The very short period of existence of the discipline limits the amount of scientific research available on the subject. The main limitation of this study is small sample size, which does not allow us to generalize our results to all combat or strength disciplines, females, junior athletes, etc.

Both groups were internally diverse due to the fact that they consisted of athletes representing different weight categories; however, statistical analysis did not show any significant differences between them in terms of body mass, height, or BMI. The structure of the groups in terms of the number of representatives of individual weight categories was similar. Although this can be considered a limitation of this study, it should be taken into account that limiting the characteristics of the group to one weight category would significantly limit the number of participants in the study, which would practically make it impossible to conduct research.

The participants were only male. Finding enough high-level female MMA athletes to establish individual research group is challenging and putting the few available female athletes in the male group would distort the results.

One of the limitations was the impossibility of directly comparing phase angle values obtained in this study with another study, which makes it hard to interpret the results because there is a lack of research analyzing solely MMA or powerlifting disciplines.

A fairly common limitation of scientific research in sport is incomplete information about test subjects’ training history. In this case, we do not know exactly how the subjects trained, nor did we know their history of nutrition and potential supplementation.

A limitation typical for research in combat and strength disciplines is the dynamic of the body mass changes during the season. The MMA athletes may present differences in body weight and body composition due to the process of getting ready for a fight, which is often associated with rapid weight loss. In research conducted by John Connor and Brendan Egan, in preparation for the fight, MMA athletes were able to lose up to 7.9% ± 3.1% of their body weight [69]. Corey A. Peacock et al. (2022) documented weight loss up to 7% within 72 h prior to the official weigh-in. The data also suggest that athletes gain nearly 10% of total weight between the official weigh-in and competition [70]. In powerlifting, there are also cases of changing weight classes by athletes to better use one’s physical potential. Due to this fact, we decided to conduct our study just after the end of the main season; it is reasonable to compare our work with other studies conducted in similar periods.

## 8. Future Research Directions

Involved in a combat sport with high intensity, MMA participants are susceptible to various injuries and trauma. Complexity and individuality alone generate need for creating well-fitting protocols, both for training and rehabilitation. Doing so requires accurate data and results. MMA is still not fully understood and needs more established referential action patterns for athletes, trainers, and physiotherapists.

The inability to find enough female participants for this study leaves space to carry on with similar research only aimed at women.

The fluctuation of anthropometric parameters depending on the phase of preparation for the fight was not examined in this study. There is necessity for further studies measuring how SMM, PBF, FM, VFM and PA alternate in pro MMA athletes while preparing for the weigh-ins and the fight afterwards.

In addition, future research could focus on investigating in more detail whether specific weight classes differ in terms of parameters such as PhA.

## Figures and Tables

**Table 1:** Results of the comparative analysis of body weight, body height, and body mass index (BMI) of the examined people practicing powerlifting (STR) and MMA.

Parameters	STR (*n* = 17)	MMA (*n* = 17)	Δ (±95% CI)	Δ %	*p* Value (*p*.adj)	ES (±95% CI)	Assess. ES
Body weight (kg)	88.4 ± 11.47	81.8 ± 10.83	6.62 (−1.18; 14.41)	8.1%	0.093 (0.280)	0.58 (−0.16; 1.25)	average
Body height (cm)	182.4 ± 6.26	181.0 ± 7.16	1.41 (−3.29; 6.11)	0.8%	0.545 (0.545)	0.21 (−0.55; 0.93)	small
BMI (kg/m^2^)	26.49 ± 2.26	24.98 ± 3.12	1.51 (−0.40; 3.42)	6.1%	0.116 (0.280)	0.54 (−0.27; 1.28)	average

Δ—absolute difference, ± 95% CI—confidence interval, Δ %—proportional change, *p* value of independent samples Students’ t-test, ES—effect size, Assess. ES—assessment of the effect size.

**Table 2:** Results of the comparative analysis of the body composition of the examined individuals practicing strength sports (GR A) and MMA (GR B).

Parameters	STR *n* = 17	MMA *n* = 17	Δ (±95% CI)	Δ %	*p* Value (*p*.adj)	ES (±95% CI)	Assess. ES
SMM (kg)	42.65 ± 5.77	39.03 ± 4.94	3.61 (−0.15; 7.37)	9.3%	0.059 (0.236)	0.66 (−0.04; 1.34)	average
PBF (%)	13.84 ± 2.49	14.68 ± 4.08	−0.84 (−3.22; 1.54)	−5.7%	0.474 (1.000)	−0.24 (−0.92; 0.44)	small
FM (kg)	12.23 ± 2.61	12.14 ± 4.11	0.09 (–2.33; 2.51)	0.8%	0.937 (1.000)	0.03 (–0.68; 0.73)	trivial
VFM (kg)	1.42 ± 0.38	1.41 ± 0.6	0.01 (−0.35; 0.36)	0.4%	0.973 (1.000)	0.01 (−0.70; 0.74)	trivial

SMM—skeletal muscle mass, PBF—percentage of body fat, FM—body fat mass, VFM—visceral fat mass.

**Table 3:** Results of the comparative analysis of the lean soft tissue mass of the subjects practicing powerlifting (STR) and mixed martial arts (MMA).

Soft Fat-Free Tissue Mass (kg)	STR *n* = 17	MMA *n* = 17	Δ (±95% PU)	Δ %	*p* Value (*p*.adj)	ES (±95% PU)	Assess. ES
Right upper limb	4.68 ± 0.76	4.21 ± 0.68	0.47 (−0.04; 0.97)	11.1%	0.068 (0.203)	0.63 (−0.09; 1.29)	average
Left upper limb	4.62 ± 0.78	4.24 ± 0.77	0.38 (−0.16; 0.92)	9.0%	0.161 (0.203)	0.48 (−0.29; 1.16)	small
Trunk	33.37 ± 4.3	30.84 ± 3.91	2.53 (−0.34; 5.41)	8.2%	0.082 (0.203)	0.60 (−0.14; 1.30)	average
Right lower limb	11.4 ± 1.61	10.34 ± 1.29	1.06 (0.04; 2.08)	10.3%	0.042 (0.169)	0.71 (−0.05; 1.39)	average
Left lower limb	11.34 ± 1.59	10.23 ± 1.18	1.10 (0.12; 2.08)	10.8%	0.029 (0.146)	0.77 (0.00; 1.45)	average

**Table 4:** Results of the comparative analysis of the fat mass of the subjects practicing powerlifting (STR) and MMA.

Fat Mass (kg)	STR *n* = 17	MMA *n* = 17	Δ (±95% CI)	Δ %	*p* Value (*p*.adj)	ES (±95% CI)	Assess. ES
Right upper limb	0.34 ± 0.18	0.41 ± 0.28	−0.06 (−0.23; 0.10)	−15.7%	0.434 (1.000)	−0.27 (−0.96; 0.42)	small
Left upper limb	0.38 ± 0.2	0.45 ± 0.29	−0.07 (−0.25; 0.10)	−16.4%	0.391 (1.000)	–0.29 (−1.01; 0.44)	small
Trunk	7.29 ± 1.48	7.18 ± 2.32	0.11 (−1.26; 1.48)	1.6%	0.866 (1.000)	0.06 (−0.64; 0.74)	trivial
Right lower limb	1.67 ± 0.35	1.66 ± 0.46	0.01 (−0.27; 0.29)	0.6%	0.943 (1.000)	0.02 (−0.68; 0.74)	trivial
Left lower limb	1.61 ± 0.35	1.53 ± 0.52	0.09 (−0.22; 0.40)	5.8%	0.565 (1.000)	0.20 (−0.52; 0.86)	trivial

**Table 5:** Results of the comparative analysis of the phase angle of the examined individuals practicing powerlifting and MMA.

Parameter	STR *n* = 17	MMA *n* = 17	Δ (±95% CI)	Δ %	*p* Value	ES (±95% CI)	Assess. ES
Phase angle (°)	8.43 ± 1.07	8.11 ± 0.62	0.32 (−0.30; 0.94)	3.9%	0.301	0.35 (−0.44; 1.05)	small

## Data Availability

Data generated in this study is available on reasonable request of a researcher from the corresponding author.
